# Monogenic Causes in Familial Stroke Across Intracerebral Hemorrhage and Ischemic Stroke Subtypes Identified by Whole-Exome Sequencing

**DOI:** 10.1007/s10571-022-01315-3

**Published:** 2022-12-29

**Authors:** Li-Hsin Chang, Nai-Fang Chi, Chun-Yu Chen, Yung-Shuan Lin, Shao-Lun Hsu, Jui-Yao Tsai, Hui-Chi Huang, Chun-Jen Lin, Chih-Ping Chung, Chien-Yi Tung, Chung-Jiuan Jeng, Yi-Chung Lee, Yo-Tsen Liu, I-Hui Lee

**Affiliations:** 1 Institute of Brain Science, National Yang Ming Chiao Tung University, Taipei, Taiwan; 2 Department of Neurology, Neurological Institute, Taipei Veterans General Hospital, No. 201, Sec. 2, Shipai Rd., Beitou District, 11217 Taipei City, Taiwan; 3 School of Medicine, National Yang Ming Chiao Tung University, Taipei, Taiwan; 4 Brain Research Center, National Yang Ming Chiao Tung University, Taipei, Taiwan; 5 Cancer Progression Research Center, National Yang Ming Chiao Tung University, Taipei, Taiwan; 6 Institute of Anatomy and Cell Biology, School of Medicine, National Yang Ming Chiao Tung University, Taipei, Taiwan

**Keywords:** Stroke, Family history, WES, Monogenic, Taiwan

## Abstract

Whole exome sequencing (WES) has been used to detect rare causative variants in neurological diseases. However, the efficacy of WES in genetic diagnosis of clinically heterogeneous familial stroke remains inconclusive. We prospectively searched for disease-causing variants in unrelated probands with defined familial stroke by candidate gene/hotspot screening and/or WES, depending on stroke subtypes and neuroimaging features at a referral center. The clinical significance of each variant was determined according to the American College of Medical Genetics guidelines. Among 161 probands (mean age at onset 53.2 ± 13.7 years; male 63.4%), 33 participants (20.5%) had been identified with 19 pathogenic/likely pathogenic variants (PVs; WES applied 152/161 = 94.4%). Across subtypes, the highest hit rate (HR) was intracerebral hemorrhage (ICH, 7/18 = 38.9%), particularly with the etiological subtype of structural vasculopathy (4/4 = 100%, PVs in *ENG, KRIT1, PKD1, RNF213*); followed by ischemic small vessel disease (SVD, 15/48 = 31.3%; PVs in *NOTCH3, HTRA1, HBB*). In contrast, large artery atherosclerosis (LAA, 4/44 = 9.1%) and cardioembolism (0/11 = 0%) had the lowest HR. *NOTCH3* was the most common causative gene (16/161 = 9.9%), presenting with multiple subtypes of SVD (*n* = 13), ICH (*n* = 2), or LAA (*n* = 1). Importantly, we disclosed two previously unreported PVs, *KRIT1* p.E379* in a familial cerebral cavernous malformation, and *F2* p.F382L in a familial cerebral venous sinus thrombosis. The contribution of monogenic etiologies was particularly high in familial ICH and SVD subtypes in our Taiwanese cohort. Utilizing subtype-guided hotspot screening and/or subsequent WES, we unraveled monogenic causes in 20.5% familial stroke probands, including 1.2% novel PVs. Genetic diagnosis may enable early diagnosis, management and lifestyle modification.

**Graphical Abstract:**

Among 161 familial stroke probands, 33 (20.5%) had been identified pathogenic or likely pathogenic monogenic variants related to stroke. The positive hit rate among all subtypes was high in intracerebral hemorrhage (ICH) and ischemic small vessel disease (SVD). Notably, two previously unreported variants, *KRIT1* p.E379* in a familial cerebral cavernous malformation and *F2* p.F382L in familial cerebral venous sinus thrombosis, were disclosed. *CVT* cerebral venous thrombosis; *HTN* Hypertensive subtype; *LAA* large artery atherosclerosis; *SV* structural vasculopathy; *U* Undetermined.

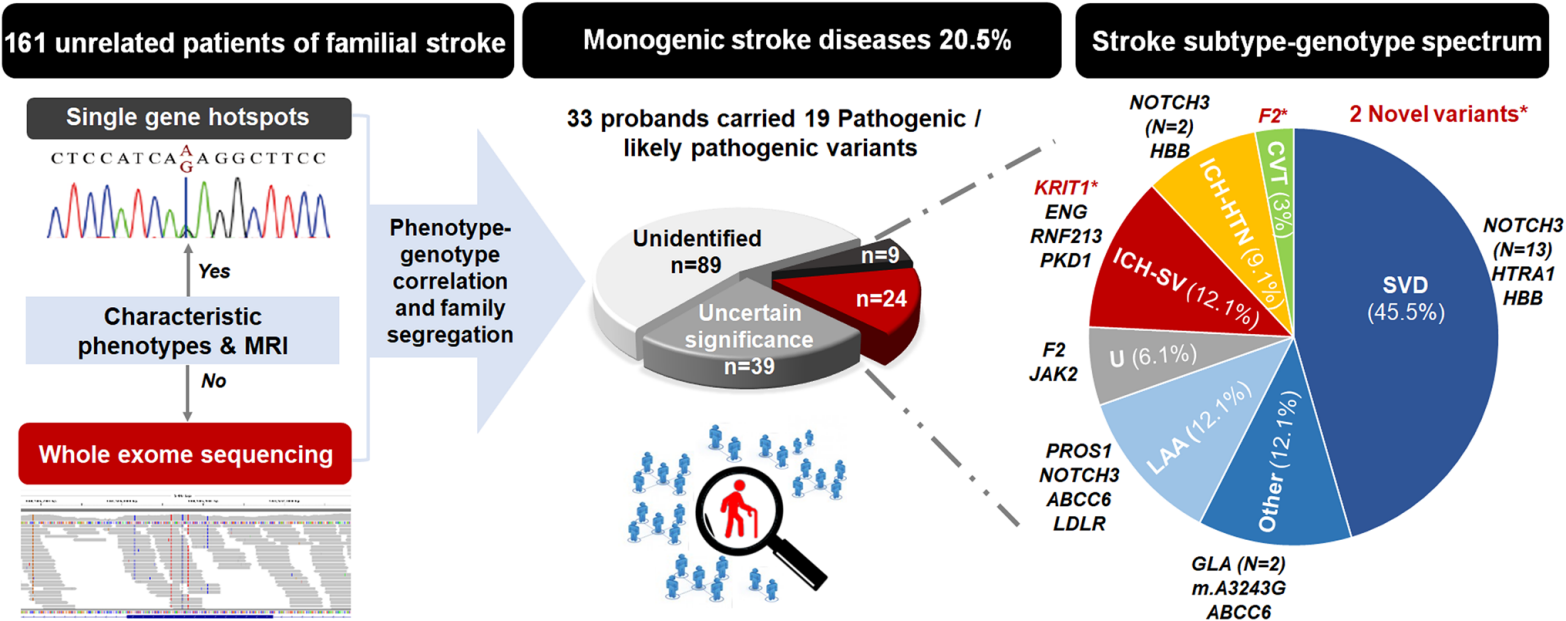

**Supplementary Information:**

The online version contains supplementary material available at 10.1007/s10571-022-01315-3.

## Introduction

Stroke is a leading cause of death and disability, which is attributed to complex genetic and environmental risk factors (Starby et al. 2014). Familial aggregation is a risk factor that increases stroke susceptibility (Chung et al. 2016; Ilinca et al. 2016). Whole-exome sequencing (WES) has become an effective tool to detect rare monogenic disorders (Rabbani et al. 2014). Lingren and his colleagues reported possibly disease-causing variants in six out of 22 familial young stroke patients (under 55 years old) by WES, revealing a higher hit rate (27.3%) than targeted gene panels (Ilinca et al. 2020). However, only a few WES studies are available addressing limited and specific cerebrovascular diseases (Santoro et al. 2018; Carrera et al. 2016; Mukawa et al. 2017; Sauvigny et al. 2020). The strategy and efficacy to apply clinical WES in familial stroke are still elusive. The main challenges come from the high heterogeneity in both phenotypes and genotypes of familial stroke patients, as well as inconsistent variant interpretation from WES data across laboratories.

There is a lack of comprehensive WES studies in Asian stroke patients. Compared to Caucasians, East Asians are more susceptible to small vessel disease (SVD), intracerebral hemorrhage (ICH), and intracranial large artery atherosclerosis (LAA) (Toyoda et al. 2015; Wei et al. 2021; Gurdasani et al. 2019; Kim and Kim 2014). Genetic variations may play an essential role in the different distribution of stroke subtypes among ethnic populations. In East Asians, cerebral autosomal dominant arteriopathy with subcortical infarcts and leukoencephalopathy (CADASIL) caused by *NOTCH3* mutations (Lee et al. 2019), CADASIL2 and CARASIL (recessive) caused by *HTRA1* mutations (Liao et al. 2015), and moyamoya disease (MD) caused by *RNF213* mutations, are particularly prevalent (Bang et al. 2020). To delineate the monogenic causes and the genotype–phenotype correlations of familial stroke in Asians, here we investigated the frequency of likely disease-causing variants across a full spectrum of stroke subtypes in a Taiwanese cohort of familial stroke patients aged 18–79 using prevalent gene/hotspot screening and/or WES, depending on stroke subtypes and neuroimaging features. The efficacy of this integrative diagnostic pipeline and the therapeutic implications for the unraveled monogenic stroke disorders were addressed.

## Materials and Methods

### Patient Enrollment and Study Design

**Fig. 1:**
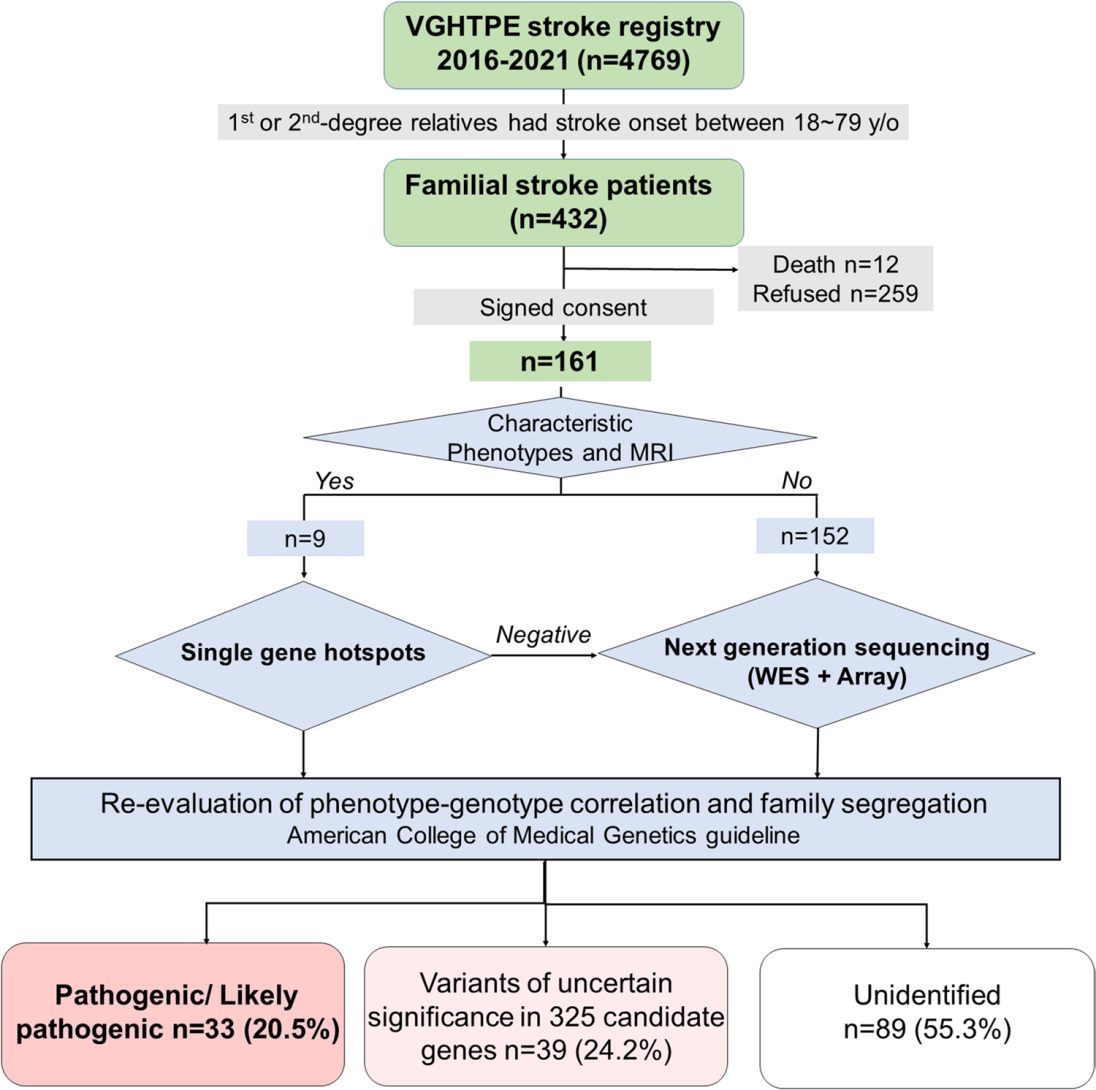
The inclusion criteria and diagnostic algorithm of familial stroke. We prospectively screened 432 patients with familial history of stroke in 4769 consecutive inpatients admitted to Taipei Veterans General Hospital (VGHTPE stroke registry) between 2016 and 2021. Totally 161 probands fulfilled the criteria and participated. Nine probands were diagnosed by sequencing specific gene hotspots and the other 152 probands were sent for whole-exome sequencing (WES) and a customized array. Thirty-three probands were identified carried pathogenic/likely pathogenic variants and 39 carried variants of uncertain significance among 325 stroke-related genes. The other 89 probands were categorized as unidentified, including 42 probands with no variants identified after variant filtering and other 47 with incompatible variants with phenotype or inheritance mode. MRI: magnetic resonance imaging

This study was performed with the approval of the Taipei Veterans General Hospital Institutional Review Board (2016-09-010C, 2020-02-007B) in accordance with the tenets of the Declaration of Helsinki. We prospectively screened 432 patients with familial ischemic or hemorrhagic stroke between September 2016 and July 2021 from 4769 inpatients consecutively admitted to Taipei Veterans General Hospital, a cohort from one of the largest tertiary referral centers in Taiwan. A positive family history was defined as a patient and at least one of the first or second-degree relatives having their first stroke between 18 and 79 years old. The inclusion criteria and diagnostic algorithm of this study are summarized in Fig. 1. The diagnosis of stroke was established by brain MRI/MRA and corresponding neurological deficits. The subtype classification was determined according to the classification of TOAST (Trial of Org 10172 Acute Stroke Treatment) for ischemic stroke (IS) and SMASH-U for intracerebral hemorrhage (ICH) (Adams et al. 1993; Meretoja et al. 2012). Cerebral venous sinus thrombosis-induced stroke was separately subtyped.

Totally, 161 probands fulfilled and provided consent to participate. Patients with characteristic clinical and neuroimaging features were first screened by conventional Sanger sequencing of specific genetic hotspots, including *NOTCH3* p.R544C for CADASIL, m.3243A > G for mitochondrial encephalopathy, lactic acidosis, and stroke-like episodes (MELAS), and the *GLA* gene for Fabry disease. The remaining undiagnosed probands were submitted to an integrative approach which combined WES and a supplementary single nucleotide polymorphism (SNP) array customized for the genomic variants of Taiwanese population, including intronic and mitochondrial variants (Axiom Genome-Wide Taiwan Biobank Array Plate, TWB 2.0 National Center for Genome Medicine, Academia Sinica, Taiwan) (Wei et al. 2021). Rare variants are defined by the minor allele frequency (MAF) equal to or less than 0.1% in the global population among gnomAD database. The MAF in local control population was obtained from Taiwan Biobank whole-genome sequencing dataset of 1517 normal subjects. The clinical significance of the genetic variant was determined according to the American College of Medical Genetics (ACMG) guideline (Kalia et al. 2017). Detailed clinical data including the medical records, intensive interviewing, laboratory, and imaging studies were reviewed by two neurologists to confirm the phenotype-genotype correlation and segregation. The level of genetic diagnosis was categorized into (1) the definite genetic diagnosis with a pathogenic/likely pathogenic variant (PV) (i.e., monogenic stroke), (2) variants of uncertain significance (VUS) in the 325 stroke-associated genes (Supplementary Table S1), and (3) unidentified (Fig. 1).

### Brain Magnetic Resonance Imaging (MRI)

All probands received MRI within 7 days after stroke onset in a 1.5 Tesla MRI scanner (Signa HDxt, GE Healthcare, Milwaukee, WI). A regular stroke MRI protocol consisted of T1-weighted imaging, T2-weighted and Fluid Attenuated Inversion Recovery (FLAIR) imaging, Diffusion-Weighted Imaging (DWI), Apparent Diffusion Coefficient (ADC), and time-of-flight (TOF) MR Angiography (MRA). For probands with SVD or ICH, T2*-weighted Susceptibility-Weighted Angiography (SWAN) was additionally applied. For probands with suspected dissection, a high-resolution contrast-enhanced vessel wall imaging was followed for intramural hematoma, intimal flap or double lumen in a 3.0 Tesla MRI scanner (Discovery MR750, GE Healthcare).

### Whole-Exome Sequencing (WES)

Genomic DNA was extracted from peripheral blood with a Gentra Puregene Blood Kit (QIAGEN, Hilden, Germany) according to the manufacturer’s instructions. Thirty-eight samples were enriched by NimbleGen SaqCap v3 (Roche NimbleGen, Inc., California, USA) and processed by the Genome Research Center of National Yang Ming Chiao Tung University, while the other 114 samples were enriched by SureSelect V6 (Agilent Technologies, California, USA) and processed by the Genomics Biotechnology Co., Ltd. All sequencing was performed on the Illumina NovaSeq 6000 platform.

### Bioinformatic Pipelines of Variant Annotation

The quality of raw reads was checked by FastQC (v0.11.4) (Andrews 2010). We used BWA (v0.7.12) (Li and Durbin 2009) and Samtools (v1.9) (Li et al. 2009) for alignment to the GRCh38 reference genome by default parameters. Marking duplications, base quality score recalibration, and variant calling were performed using GATK (v4.1.4.1) (McKenna et al. 2010; Van der Auwera et al. 2013). Variants with read depths less than 30 were excluded from data interpretation. After variant calling, multiple reference databases, including ClinVar (retrieved 2021-07), dbNSFP (v4.1a), dbSNP (v154), and Online Mendelian Inheritance in Man (OMIM, retrieved 2021-07), were applied for further annotation (McLaren et al. 2016).

### Variant Filtration and Prioritization of Candidate Causal Variants

We first searched for stroke-related pathogenic and likely pathogenic variants (PVs) in the ClinVar database (https://www.ncbi.nlm.nih.gov/clinvar/). The stroke-related phenotypes were determined according to the OMIM database (https://www.omim.org/). If no reported or phenotype-compatible PV was found, we then examined novel rare variants which were predicted to cause structural and functional impacts on the coding products in 325 stroke-related genes (Supplementary Table S1) by searching the terms of stroke, ischemic stroke, hemorrhagic stroke, stroke-like episode, cerebral hemorrhage, lacunar stroke, and thromboembolic stroke in OMIM and PubMed (Ilinca et al. 2019; Hamosh et al. 2005; Dichgans et al. 2019). The selected variants must have met all of the following inclusion criteria: (1) the global MAF ≤ 0.1% in public genome database; (2) not reported as a benign or likely benign variants in ClinVar or SNP database; (3) relatively evolutionarily conserved (GREP +  +  > 3); (4) computational predictions assessed by at least two scoring systems (SIFT, Polyphen2, MutationTaster, computer-aided design and drafting score > 20) or Emsembl rare exome variant ensemble learner score ≥ 0.6 (Ioannidis et al. 2016); (5) considered phenotype-genotype correlation. If the selected variant fulfilled pathogenic or likely pathogenic according to the ACMG guideline (PVs, judged with InterVar’s assistance) (Kalia et al. 2017; Li and Wang 2017), Sanger sequencing validation and intrafamilial segregation were performed in the proband and available family members.

### Statistical Analysis

The statistical analysis was conducted using SPSS (Version 20.0. for Windows, Armonk, NY: IBM Corp. USA). To compare the characteristics between probands with and without monogenic PVs, two-sample independent t-tests were used for age at onset, initial severity of National Institute of Health Stroke Scale (NIHSS) scores, modified Rankin Scale (mRS) scores at baseline and three months after stroke, and risk factors. Risk factor numbers (0–7) were derived by summing the risk factors present in a stroke patient, including hypertension, diabetes, hyperlipidemia, atrial fibrillation, cigarette smoking, excessive alcohol consumption, and obesity (body mass index ≥ 30). Categorical variables, including sex, stroke subtypes, and obesity, were compared by the chi-square test or Fisher’s exact test. The hit rate of monogenic stroke based on stroke subtyping was defined as the patient number with monogenic causes divided by the total patient number of that specific subtype. Statistical significance was set at *p* < 0.05.

## Results

### Patient Characteristics of the Familial Stroke Caused by Monogenic Etiologies

Nine out of 161 patients (9/161 = 5.6%) having characteristic phenotypes and brain MRI/MRA were diagnosed via conventional Sanger sequencing of specific gene hotspots, including six with *NOTCH3*-related CADASIL (all carried p.R544C), two with *GLA*-related Fabry disease (p.Y365* and IVS4 + 919G > A), and one with m.3243A > G-related MELAS. For the remaining 152 probands who had a family history but the candidate genes were unclear (152/161 = 94.4%), WES revealed pathogenic/likely pathogenic variants (PVs) in 24 individuals (24/152 = 15.8%). The overall yield rate to detect a PV for relevant phenotype by the combination of WES and conventional sequencing was high, 20.5% (33/161). The clinical diseases and the genotypes of the 33 probands with ascertained monogenic stroke are summarized in Table 1. The detailed phenotypic and genotypic spectrum was described as follows. Another 23 family members ascertained with monogenic strokes shared the corresponding phenotypes and genotypes with individual probands (Supplementary Fig. S1-S2).

**Table 1:** Genotypes and phenotypes of the 33 unrelated probands diagnosed as monogenic familial stroke, including two carrying novel variants^a^

Diseases	Stroke subtype	Gene	Variants	Inheritance	ClinVar	ACMG (Classification)	SNP ID
Ischemic Stroke							
CADASIL	SVD (*n = *12, 1 Hom), LAA (*n = *1),	*NOTCH3*	p.R544C	AD	Pathogenic	Pathogenic	rs201118034
	SVD (*n = *1)	*NOTCH3*	p.R1231C	AD	Conflicting	Likely pathogenic (PM1 + PP2 + PP3 + PP4 + PP5)	rs201680145
CADASIL2	SVD (*n = *1)	*HTRA1*	p.G276A	AD	Likely pathogenic	Likely pathogenic	rs1554952277
Fabry disease	Other (*n = *2)	*GLA*	p.Y365*	XLR	Pathogenic	Pathogenic	rs104894849
		*GLA*	IVS4 + 919G > A	XLR	Pathogenic	Pathogenic	rs199473684
Familial hyperlipidemia	LAA (*n = *1)	*LDLR*	p.D90N	AD	Conflicting	Likely pathogenic (PM1 + PM5 + PP2 + PP3)	rs749038326
MELAS	Other (*n = *1)	*mtDNA*	m.3243A > G	Maternal	Pathogenic	Pathogenic	rs199474657
Protein S deficiency	LAA (*n = *1)	*PROS1*	p.Y592*	AD	Pathogenic	Pathogenic	rs199469503
Myeloproliferative neoplasia	Undetermined (*n = *1)	*JAK2*	p.V617F	AD	Pathogenic	Pathogenic	rs77375493
Pseudoxanthoma elasticum	LAA (*n = *1, Het)	*ABCC6*	p.R1235W	AD	Likely pathogenic	Likely pathogenic	rs63750402
	Other (*n = *1, Hom)	*ABCC6*	p.E709K	AR	Variant of uncertain significance	Likely pathogenic (PM1 + PM2 + PP3 + PP4)	rs114303883
Thalassemia	SVD (*n = *1)	*HBB*	p.F42fs	AD	Pathogenic	Pathogenic	rs80356821
Coagulation factor II disorder	Undetermined (*n = *1)	*F2*	p.R596Q	AD	Pathogenic	Pathogenic	rs387907201
Intracerebral Hemorrhage							
CADASIL	HTN (*n = *2)	*NOTCH3*	p.R544C	AD	Pathogenic	Pathogenic	rs201118034
Cerebral cavernous malformation^a^	SV (*n = *1)	*KRIT1*	p.E379*	AD	Not reported	Pathogenic (PVS1 + PM2 + PP3 + PP4)	Novel
Hereditary hemorrhagic telangiectasia	SV (*n = *1)	*ENG*	p.Y258Lfs*76	AD	Pathogenic	Pathogenic	rs1588581902
Moyamoya disease	SV (*n = *1)	*RNF213*	p.R4810K	AD	Likely pathogenic	Likely pathogenic	rs112735431
PKD	SV (*n = *1)	*PKD1*	c.11014-10C > A	AD	Pathogenic	Pathogenic	rs555703777
Thalassemia	HTN (*n = *1)	*HBB*	p.K18*	AD	Pathogenic	Pathogenic	rs33986703
Cerebral venous sinus thrombosis							
Coagulation factor II disorder^a^	Hemorrhagic infarction (*n = *1)	*F2*	p.F382L	AD	Not reported	Likely pathogenic (PM1 + PM2 + PP1 + PP2 + PP4)	Novel

*ACMG* American College of Medical Genetics, *AD* autosomal dominant, *AR* autosomal recessive, *CADASIL* Cerebral autosomal dominant arteriopathy with subcortical infarcts and leukoencephalopathy, *ClinVar* https://www.ncbi.nlm.nih.gov/clinvar/, *Het* Heterozygosity, *Hom* Homozygosity, *HTN* hypertensive subtype, *ICH* Intracerebral hemorrhage, *LAA* Large artery atherosclerosis, *MELAS* Mitochondrial encephalopathy, lactic acidosis, and stroke-like episodes, *PKD* Polycystic kidney disease, *PM* Pathogenic moderate, *PS* Pathogenic supporting, *PVS* Pathogenic very strong, *SV* Structural vasculopathy, *SVD* Small vessel disease, *VUS* Variants of uncertain significance, *XLR* x-link recessive

^a^Novel variant identified

The demographic characteristics of 161 probands are shown in Table 2. The mean age at onset was 53.2 ± 13.7 years old and 63.4% were male. The most common ischemic stroke subtype was SVD (29.8%) and LAA (27.3%). ICH accounted for 11.3%. The stroke subtype distribution was close to previous publications for Asians (Kim and Kim 2014). Notably, the subtype distribution was different between the patients with and without ascertained monogenic stroke. The patients ascertained with monogenic stroke (*n* = 33) appeared to have more SVD (45.5% vs 25.8%, *p* = 0.046) and ICH (21.2% vs 8.6%, *p* = 0.04) as compared to those unassigned probands. In contrast, LAA and cardioembolism were the less common subtypes in the monogenic group. Additionally, the patients with monogenic stroke had fewer vascular risk factors (the proportion with 0–1 risk factor: 42.4% vs 19.5%, *p* = 0.01). Apart from stroke subtypes and risk factors, we did not find significant differences in the other clinical features between the two groups, including age at onset, sex, the number of strokes affected members per family cluster, the initial severity of National Institute of Health Stroke Scale (NIHSS) and the modified Rankin Scale (mRS) outcome at three months after stroke.

**Table 2:** Demographics of ascertained monogenic versus unassigned familial stroke patients

Clinical features	Total	Monogenic stroke	Unassigned	*p*-value
Patient number	161	33 (20.5%)	128 (79.5%)	N/A
Age at onset (y)	53.2 ± 13.7	51.5 ± 14.5	53.7 ± 13.5	0.43
Age ≤ 55, n (%)	94 (58.4)	19 (57.6)	75 (58.6)	0.91
Sex, Men (%)	63.4	60.6	63.6	0.66
Stroke affected members per family cluster	3.1 ± 1.4	3.0 ± 1.0	3.1 ± 1.5	0.5
Infarction subtype (%)	142 (88.2%)	25 (75.8%)	117(90.7%)	0.08
Small vessel disease	48 (29.8%)	15 (45.5%)	33 (25.8%)	0.046*
Large artery atherosclerosis	44 (27.3%)	4 (12.1%)	40 (31.2%)	0.047*
Undetermined	18 (11.2%)	2 (6.1%)	16 (12.5%)	0.54
Other determined	21 (13%)	4 (12.1%)	17 (13.3%)	0.99
Cardiac embolism	11 (6.8%)	0 (0%)	11 (8.6%)	N/A
Intracerebral hemorrhage	18 (11.3%)	7 (21.2%)	11 (8.6%)	0.04*
Cerebral venous thrombosis	1 (0.6%)	1 (3%)	0 (0%)	N/A
Risk factor numbers (0–7)	2.2 ± 1.2	1.8 ± 1.4	2.4 ± 1.1	0.03*
Risk factors ≤ 1	39 (24%)	14 (42.4%)	25 (19.5%)	0.01*
Initial severity NIHSS	6.2 ± 7.4	7.3 ± 8.3	6 ± 7.2	0.43
3-month favorable outcome	108 (67.1%)	24 (72.7%)	84 (65.6%)	0.57

All continuous variables were presented as mean ± standard deviation

*NIHSS* National Institute of Health Stroke Scale; *mRS* Modified Rankin Scale at 3 months. Favorable outcome as mRS 0–2. Risk factors included hypertension, diabetes mellitus, hyperlipidemia, atrial fibrillation, cigarette smoking, excessive alcohol consumption, and obesity (body mass index ≥ 30).

*p < 0.05 considered significant for monogenic stroke vs. non-monogenic case

### Phenotypic and Genotypic Spectrum and the Hit Rate of Monogenic Causes

Among the 33 probands with ascertained monogenic stroke (Table 1), CADASIL was the most common hereditary stroke disease (*n* = 16, 48.5%). The other disorders included pseudoxanthoma elasticum (*n* = 2), coagulation factor II disorder (*n* = 2), beta-thalassemia (*n* = 2), Fabry disease (*n* = 2), MELAS (*n* = 1 for each disease below), protein S deficiency, hereditary hemorrhagic telangiectasia (HHT), polycystic kidney disease (PKD), CADASIL2, moyamoya disease (MD), cerebral cavernous malformation (CCM), familial hyperlipidemia, and primary thrombocythemia.

**Fig. 2:**
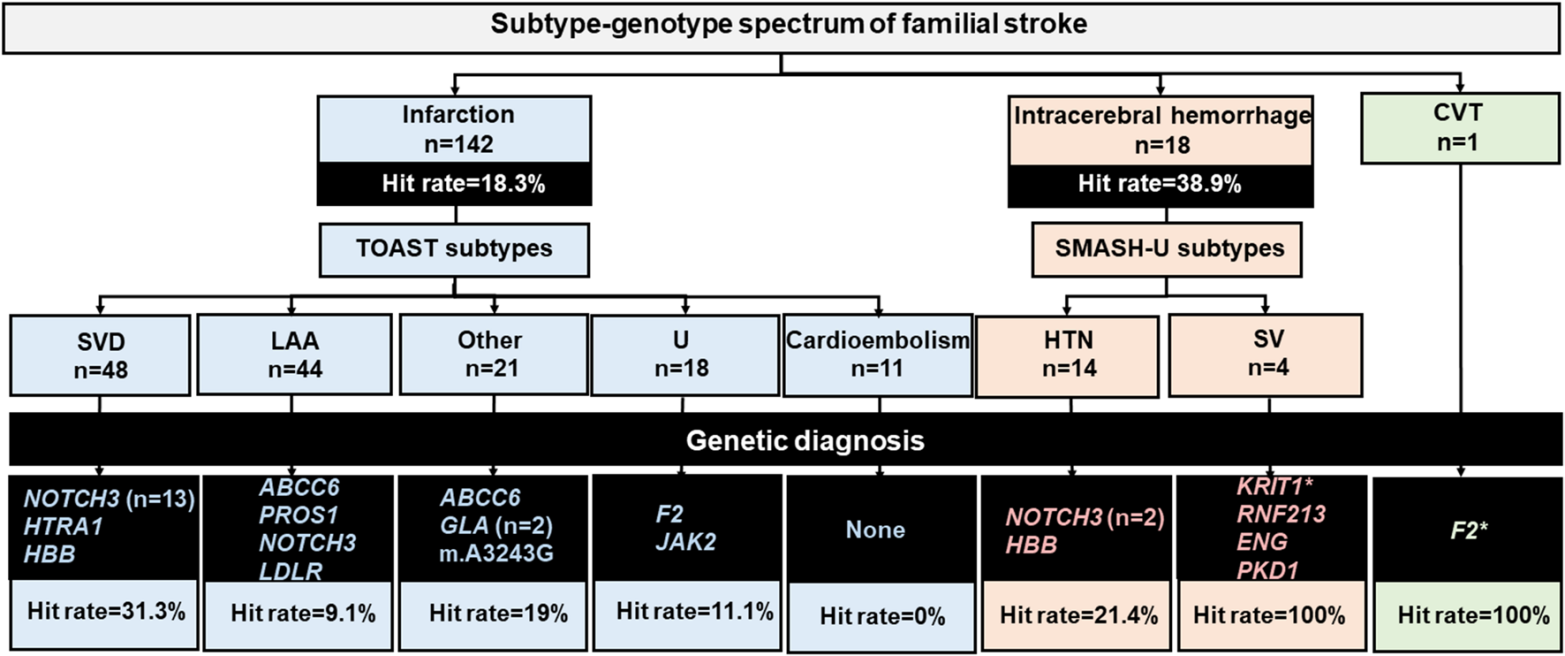
Phenotypic and genotypic spectrum and the hit rate across stroke subtypes. *CVT* Cerebral venous sinus thrombosis, *HTN* hypertensive subtype, *LAA* large artery atherosclerosis, *SMASH*-U Structural lesion, Medication, Amyloid angiopathy, Systemic/other disease, Hypertension, Undetermined, *SV* structural vasculopathy, *SVD* small vessel disease, *TOAST* Trial of Org 10172 in Acute Stroke Treatment. *Novel variant

The hit rates (HR) of detecting causative genes for a specific subtype are shown in Fig. 2. The greatest diagnostic yield was in ICH patients (7/18, 38.9%). Specifically, all four patients with structural vasculopathy subtype had a monogenic etiology (4/4, 100%): *KRIT1* for CCM, *RNF213* for MD, *ENG* for HHT, and *PKD1* for PKD. For ischemic stroke (IS), the SVD subtype had the highest HR (15/48, 31.3%), followed by other determined causes (4/21, 19%), whereas the LAA (4/44, 9.1%) and the cardioembolic (0%) subtypes had the lowest HR. *NOTCH3* mutations presented with various subtypes, including SVD (*n* = 13), hypertensive ICH (*n* = 2), and LAA (*n* = 1).

#### Intracerebral Hemorrhage (ICH)

**Fig. 3:**
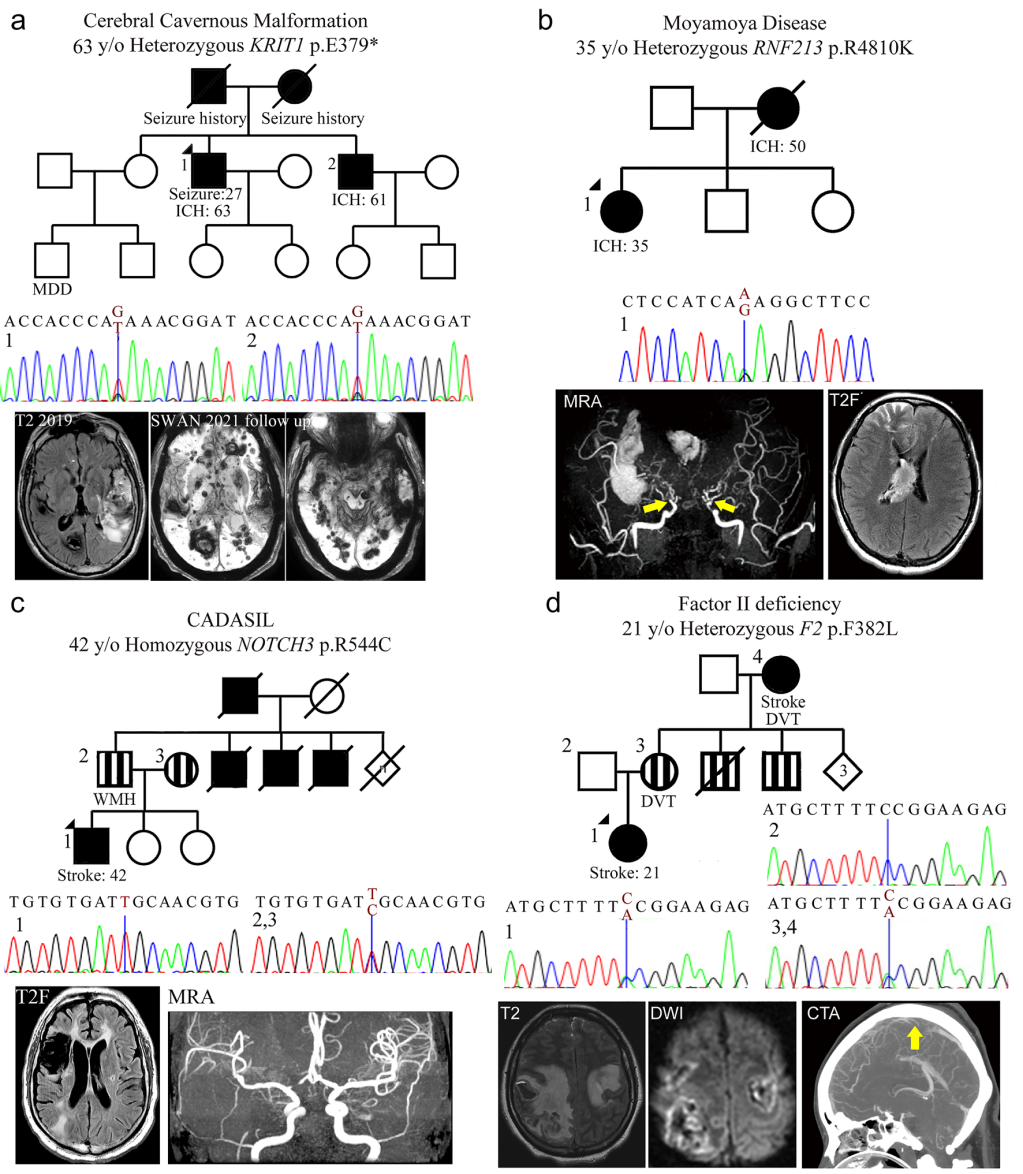
Representative pedigrees of monogenic familial strokes. **a** A 63-year-old man with numerous cerebral cavernous malformations carried a novel stop-gain variant *KRIT1* p.E379*, which was segregated in terms of genotype and phenotype with his younger brother. **b** A 35-year-old woman with intraventricular hemorrhage due to moyamoya disease carried a heterozygous *RNF213* p.R4810K variant. **c** A 42-year-old man with cerebral autosomal dominant arteriopathy with subcortical infarcts and leukoencephalopathy (CADASIL) with recurrent infarctions and severe white matter hyperintensities carried homozygous *NOTCH3* p.R544C variants, which was segregated with his parents. **d** A 21-year-old woman with superior sagittal venous sinus thrombosis and bilateral hemorrhagic infarctions carried a novel likely pathogenic variant *F2* p.F382L, which was segregated within her family. *AO* age of onset, *CTA* Computed tomography angiography, *DVT* deep vein thrombosis, *DWI* diffuse weighted image, *MRA* magnetic resonance angiography, *SWAN* Susceptibility weighted angiography, *WMH* white matter hyperintensity. Yellow arrowheads indicate the occlusive vessel

The representative monogenic cases identified using WES are shown in Fig. 3. We disclosed a novel stop-gain variant, *KRIT1* p.E379* in a 63-year-old man with multiple CCMs presented as recurrent ICH and cavernoma-related epilepsy (Fig. 3a). His epilepsy was firstly diagnosed at 27 years old and poorly controlled by medication since then. After his first ICH event in 2019, multiple CCMs had been found by MRI and the largest (4.8 cm) cavernoma at the left temporal region was removed by craniotomy. The histopathology showed aggregated dilated vascular space with thinning wall and thrombus formation, which was compatible with cavernoma. His younger brother, who carried the same variant, also had recurrent ICH due to multiple CCMs and microbleeds in the bilateral cerebral and cerebellar hemispheres. The segregation analysis of *KRIT1* p.E379* was consistent with the disease and only in two affected family members. This novel variant is absent in global population database gnomAD and Taiwan Biobank database. The stop-gain variant is presumed to cause loss of function of the gene at vascular endothelial tight junctions (Merello et al. 2016). It is predicted to be deleterious by multiple lines of bioinformatic evidence (CADD phred score 40, GERP +  + NS 5.66). Therefore, this variant is considered pathogenic according to ACMG guideline criteria (PVS1 + PM2 + PP3 + PP4, see Table 1). Based on the genetic diagnosis, we provided genetic counseling, close follow-up of brain MRI, and strict blood pressure control for the patients and affected family members.

A 35-year-old woman with no known risk factor presented by acute intraventricular hemorrhage and obstructive hydrocephalus, and the brain MRA documented Suzuki stage IV with occlusive changes in the bilateral distal internal carotid arteries and tenuous anterior and middle cerebral arteries (Fig. 3b). Her mother expired due to a hemorrhagic stroke in her late 40 s. She was identified to carry a heterozygous *RNF213* p.R4810K variant, a hotspot variant of Moyamoya disease (MD) in the East Asia population (Lee et al. 2015; Ishigami et al. 2022). We provided education about the condition and transferred to neurosurgery department for extracranial-intracranial arterial bypass according to treatment recommendations (Fujimura et al. 2022).

WES detected an intronic pathogenic variant *PKD1* c.11014-10C > A in a 38-year-old man who suffered from subarachnoid hemorrhage by a ruptured anterior communicating artery aneurysm and PKD. His father had PKD and died from a ruptured brain aneurysm. All his three siblings had been diagnosed as PKD and shared the same *PKD1* variant. Computed tomography (CT) angiography was suggested for these siblings to detect asymptomatic brain aneurysms. Also, a 23-year-old woman who had arteriovenous fistula presented by ICH and seizure had been identified *ENG* p.Y258Lfs*76. The proband and both of her two siblings had spontaneous and recurrent nosebleeds since childhood, which was compatible with hereditary hemorrhagic telangiectasia (HHT) phenotype. Education was also provided for these two probands.

Another two probands carried the CADASIL hotspot PV *NOTCH3* p.R544C and suffered from dementia prior to the ICH event. *HBB* p.K18* variant was detected in a 63-year-old man who suffered from hypertensive thalamic ICH and thalassemia (low hemoglobin).

#### Ischemic Small Vessel Disease (SVD)

In this subtype, twelve probands were identified as carrying the hotspot PV *NOTCH3* p.R544C and one as carrying the *NOTCH3* p.R1321C. A representative proband, carrying the homozygous *NOTCH3* p.R544C variants (Fig. 3c), had his first stroke at 42 years old and later had early-onset dementia (Mini-Mental State Examination = 21) at the age of 46. His brain MRI/MRA revealed large tissue loss in the right anterior frontal region, bilateral severe white matter hyperintensities and multisegmental mild narrowing in the M1 segment of right middle cerebral artery. Also, he had post-stroke seizure under Frisium and Depakine. Both of his parents were confirmed carrying heterozygous *NOTCH3* p.R544C with moderate white matter hyperintensities. Regular MRI follow-up for brain microbleeds and careful antithrombotic adjustment were suggested. The other pedigrees of *NOTCH3*-related stroke were presented in Supplementary Fig. S1. Another 51-year-old woman with CADASIL syndrome of stroke and mild dementia carried heterozygous *HTRA1* p.G276A, which was reported to be likely pathogenic to cerebral autosomal dominant arteriopathy with subcortical infarcts and leukoencephalopathy type 2 (CADASIL2) (Lee et al. 2018). Furthermore, a 52-year-old woman who carried *HBB* p.F42fs variant and who suffered from a lacunar stroke in the motor area with thalassemia.

#### Cerebral Venous Sinus Thrombosis (CVT)

We discovered a novel variant *F2* p.F382L in a 21-year-old woman suffering from acute consciousness change and generalized tonic–clonic seizures due to cerebral venous sinus thrombosis and hemorrhagic infarction at the bilateral frontoparietal cortices (Fig. 3d). Her mother and maternal grandmother, who also carried *F2* p.F382L, both suffered from recurrent episodes of deep vein thrombosis and cerebral venous thrombosis. One maternal uncle died from pulmonary embolism at his 30 s and another suffer from stroke and deep vein thrombosis at his 50 s. Decreased coagulation factor II activities were observed in the proband, her mother, and grandmother (44.7, 67.1, and 68.5%, respectively; reference: 79–131%). The segregation analysis was consistent with the disease in the aforementioned three affected family members and one unaffected family member. This novel variant is absent in global population database gnomAD and Taiwan Biobank database. However, it is unknown how this mutation leads to low factor II activities and prothrombotic and prohemorrhagic tendency. The variant location is highly evolutionary conserved (GERP +  + NS = 5.23, phastCons = 1) for encoding factor IIa thrombin and fibrin interaction exosite I domain (Huntington 2005). Missense variants in the *F2* gene have very low rate as benign variants (ClinVar *F2* missense variants: 21/38 pathogenic or likely pathogenic, while 2/38 benign or likely benign). Taken together, this variant is considered likely pathogenic according to ACMG guideline criteria (PM1 + PM2 + PP1 + PP2 + PP4, see Table 1). The establishment of genetic etiology introduced timely non-vitamin-K oral anticoagulant therapy with a specific factor X inhibitor (Yaghi et al. 2022), which successfully prevented the recurrence of thromboembolic events and achieved good functional outcomes in this family for years of followed-ups till now.

#### Other Determined Diseases and Undetermined Ischemic Stroke

We found a 43-year-old man proband carrying a rare homozygous *ABCC6* p.E709K variant had infarcts at the bilateral corona radiata, bilateral basal ganglia, and pons because of vertebrobasilar artery dissection, which was phenotypic compatible with ABCC6 related pseudoxanthoma elasticum. Pseudoxanthoma elasticum is an inherited elastic fiber disorder usually caused by *ABCC6* variants in an autosomal recessive inheritance pattern, but heterozygous carriers may express partial phenotypes (Miksch et al. 2005; Plomp et al. 2004). Besides, a rare pathogenic variant, *F2* p.R596Q, known for thrombophilia and antithrombin resistance (Takagi et al. 2016), was identified in a 41-year-old man presented by embolic stroke in the right parietal cortex. The proband’s factor II activity remained mildly low during follow-ups. Lastly, a well-known pathogenic variant *JAK2* p.V617F for the myeloproliferative disorder was identified in a 72-year-old woman with thrombocythemia and an undetermined subtype of stroke.

#### Large Artery Atherosclerosis (LAA)

We found a 59-year-old man proband who had basilar artery atherosclerotic infarction as well as peripheral artery occlusive disease, carrying the heterozygous *ABCC6* p.R1235W variant, which is known to be pathogenic for pseudoxanthoma elasticum. Also, *LDLR* p.D90N, a PV to familial hyperlipidemia, was detected in a 55-year-old man suffered from recurrent stroke with the third event of an acute vertebral artery occlusion infarction over the bilateral cerebellum and left medulla oblongata. Furthermore, WES identified *PROS1* p.Y592* mutation in a 72-year-old man who had protein S deficiency and episodes of large artery atherosclerosis-related infarction and deep vein thrombosis. The same variant was also detected in his son who suffered from deep vein thrombosis and decreased protein S activity as well. Lastly, a 62-year-old woman suffered from right posterior cerebral artery in-situ thrombosis infarction was identified carrying the hotspot pathogenic variant *NOTCH3* p.R544C.

### Stroke-Associated Variants of Uncertain Significance (VUS)

Apart from pathogenic or likely pathogenic findings, 41 rare VUS (Table 3) in 325 stroke-related candidate genes (Supplementary Table S1) were detected in 39 probands, including eight novel variants: *ABCA1* p.H1223P, *ACTA2* p.N298S, *COL5A1* p.P930R, *FBN1* p.Q514E, *FBN1* p.K1052T, *GP1BA* p.P437*, *RNF213* p.N1631Efs*34, and *TSC1* p.P397del. Although the evidence of pathogenicity is not enough due to the unavailability of familial segregation or the paucity of their functional impacts currently, the rare allele frequency in our population, in silico prediction and the corresponded clinical manifestations indicate that these variants may still be potentially associated with the pathogenesis of stroke. We would like to report them here and expect more evidence to elucidate their clinical significance in future. Finally, we identified no findings after variant filtering in 42 probands and the other 47 had incompatible variants with phenotype or inheritance mode, they were categorized as unidentified (*n* = 89).

**Table 3:** Variants of uncertain significance in 325 stroke-associated genes detected in this study

Gene	Disease	Variants	SNP ID	ACMG
*ABCA1*	Familial HDL deficiency	c.A3668C: p.H1223P	Novel	PM2
		c.G5140A: p.A1714T	rs758640488	PM2, PP3
*ACTA2*	Moyamoya disease	c.A893G:p.N298S	Novel	PM2,PP2,PP3,PP4
*ACVRL1*	Hereditary Hemorrhagic Telangiectasia	c.G1444A:p.A482T	rs777374619	PM1,PM2,PP3
*ANGPTL6*	Familial intracranial aneurysms	c.1003dup: p.Y335Lfs*10	rs754920128	PM2
*APP*	Cerebral amyloid angiopathy	c.A143G:p.Q48R	rs1387322785	PM1, PM2, PP3
*COL1A1*	Ehlers-Danlos syndrome	c.G344A:pG115E	rs767924851	PP2, PP3, BP1
*COL4A2*	PADMAL	c.G2954T:p.G985V	rs769851485	PP3
		c.C4514T:p.P1505L	rs777086508	PM1,PM2,PP3,PP4
*COL5A1*	Ehlers-Danlos syndrome	c.C2789G:p.P930R	Novel	PM2, PP3
*COL5A2*		c.C382T:p.R128C	rs1247714244	PM2, PP3
		c.C3038T:p.A1013V	rs372220538	PP3
*F2*	Coagulation factors deficiency	c.C1366T:p.R456W	rs139552841	PM1,PM2,PP2
*F5*		c.C4949T:p.A1650V	rs753691316	PM1,PM2,PP3
*F11*		c.C1107A: p.Y369*	rs773905328	PVS1, PP4, PP5
*FBN1*	Marfan syndrome	c.G763A:p.G255R	rs781113082	PM1,PM2,PP3,PP4
		c.C1540G:p.Q514E	Novel	PM2
		c.A3155C:p.K1052T	Novel	PM1, PM2
*FLNC*	Cardiomyopathy	c.C1205T:p.T402I	rs374757755	PM2, PP2, PP3
*GP1BA*	Bernard-Soulier syndrome	c.1275_1278del: p.T427Qfs*44	rs763460861	PVS1, PM2
		c.A1311*: p.P437*	Novel	PM2
*JAK2*	Chronic myeloproliferative diseases	c.C1141T:p.H381Y	rs774526479	PM1,PP3,PP4
*KCNA5*	Familial atrial fibrillation	c.213_245dup: p.D72_P82dup	rs144879674	PM4, PP4
*LDLR*	Familial hypercholesterolemia,	c.G409A: p.G 137S	rs730882082	PM2, PP3, PP5
*MYBPC3*	Cardiomyopathy	c.G104A: p.R35Q	rs397515885	PM2, PP2, BP1
*MYH6*	Cardiomyopathy	c.G5696A:p.R1899H	rs61731171	PP2, PP3
*MYLK*	Aortic aneurysm / arterial dissection	c.G3379C:E1127Q	rs777401981	PM2, PP2, PP3
		c.G4528A: p.D1510N	rs1483753716	PM2, PP2, PP3
*NBEAL2*	Gray platelet syndrome (from same proband)	c.G211A: p.A71T	Novel	PM2, PP3, PP4
		c.C7351T: p.R2451W	rs748364740	
		c.C7486T: p.R2496W	rs754133402	
*NOTCH3*	CADASIL	c.G391C:p.G131R	rs767150916	PM1, PM2, PP3
*PKD1*	Polycystic kidney disease	c.C6070T: p.R2024C	rs199943712	PP3, BP1
*RNF213*	Moyamoya disease	c.4890dup:p.N1631Efs*34	Novel	PM2
		c.T11668C:p.S3890P	rs1203492091	PM2, BP4
		c.G12913T: p.V4305L	rs1399368898	PM2
		c.C13726T:p.P4576S	rs776390324	PP3
*SCN2B*	Familial atrial fibrillation	c.G71-1A: p.?	rs758825110	PP3
*TGFBR1*	Loeys-Dietz syndrome	c.A491G:p.N164S	rs767785290	PM2, PP4
*TREX1*	RVCL	c.294dup:p.C99Mfs*3	rs760594164	PM1
*TSC1*	Tuberous sclerosis	c.1188_1190del: p.P397del	Novel	PM2, PM4, PP4, BP3

*ACMG* American College of Medical Genetics, *CADASIL* cerebral autosomal dominant arteriopathy with subcortical infarcts and leukoencephalopathy, *HDL* High-density lipoprotein, *OMIM* Mendelian Inheritance in Man, *PADMAL* pontine autosomal dominant microangiopathy and leukoencephalopathy, *PM* Moderate evidence of pathogenicity, *PP* Supporting evidence of pathogenicity, *PVS* Very strong evidence of pathogenicity, *RVCL* Retinal Vasculopathy with Cerebral Leukoencephalopathy

## Discussion

We proposed a stroke subtype-guided WES in combination with prevalent gene/hotspot screening and achieved an overall diagnostic yield 20.5% of monogenic causes in 33 out of 161 unrelated probands with well-defined familial stroke. This study included all IS and ICH subtypes, and thus outlined a more comprehensive genomic landscape of familial stroke. The identified PVs were distributed in a wide range of genes and rare diseases. For example, CADASIL syndrome could be caused by *NOTCH3* or *HTRA1* mutations, both account for a significant number of SVD and/or ICH patients in our population (Lee et al. 2018). Notably, we identified two novel PVs, *KRIT1* p.E379* in a family of cerebral cavernous malformation-related ICH, and *F2* p.F382L in a family of cerebral venous thrombosis-related stroke, respectively. Our findings demonstrated the power and necessity of WES in genetic diagnosis of monogenic stroke, a disease entity with high heterogeneity and complex etiologies.

Compared with the previous next-generation sequencing (NGS) studies in stroke patients, our diagnostic yield was relatively high as summarized in Table 4, although these study designs, methods and key findings were substantially different. Overall, most NGS studies were limited by small sizes, restricted screened genes, or narrow stroke subtypes (Tan et al. 2019; Lee et al. 2019; Bersano et al. 2016; Ilinca et al. 2020; Mishra et al. 2019; Auer et al. 2015). According to our results, the subtypes of familial ICH and SVD stroke, determined by the clinical features and characteristic brain MRI features, can provide the most informative clue to possible underlying monogenic etiologies. The highest HR was in the patients with the ICH (38.9%), especially for structural vasculopathy (100%), followed by SVD (31.3%). In parallel, when a patient with familial stroke has very few (0–1) conventional vascular risk factors, an underlying monogenic etiology should be aggressively searched. Otherwise, there was no difference in age at onset, sex, family history, the initial stroke severity or stroke outcome between the patients with and without a definite genetic diagnosis.

**Table 4:** Comparison of the diagnostic yield of monogenic stroke diseases among previously relevant genetic studies and our study*

Study	Study design and inclusion criteria (n)	Nation	Sequencing method	Diagnostic yield of monogenic stroke diseases
Chang et al.*	Familial stroke including ischemic and intracerebral hemorrhage, onset 18–79 y/o (*n = *161)	Taiwan	WES (VUS in 325 genes) + Targeted array	Overall 20.5%; ICH: 35.3%; SVD: 31.3%; O: 22.7%; U: 11.1%, LAA: 9.1%
Ilinca et al 2020	Familial stroke, onset ≤ 55 y/o (*n = *22)	Sweden, Finland	WES (VUS in 254 genes)	Overall 27.3%; ICH: 1/1; CE: 1/2; SVD: 3/11; U: 1/4
Mishra et al 2019	Case–control design, cohort of noninstitutionalized stroke ≤ 80 y/o (*n = *1469)	France	WES (VUS in 5 genes)	SVD: 0.8%
Tan et al 2019	MRI confirmed small vessel disease stroke, onset ≤ 70 y/o (*n = *950)	U.K	Targeted array (15 genes)	1.5%
Lee et al 2019	Consecutive ischemic stroke patients (*n = *800) versus 7038 healthy controls	Taiwan	Single-gene sequencing of *NOTCH*3	6.5% in SVD; 0.9% in healthy controls
Bersano et al 2016	Suspected monogenic diseases, fulfilled at least one of the following criteria: 1. Unknown cause, < 3 conventional vascular risk factors 2. Stroke onset ≤ 55 y/o 3. Positive familial history, 4. ≥ 2 associated clinical features (*n = *227)	Italy	Sanger sequencing of the hotspots for 5 diseases (CADASIL, Fabry disease, MELAS, CAA, Marfan syndrome)	Overall 7% CADASIL:9/103 Fabry: 1/33 CAA: 1/70 MELAS: 2/16 Marfan: 1/5

*CADASIL* Cerebral autosomal dominant arteriopathy with subcortical infarcts and leukoencephalopathy, *CAA* Cerebral amyloid angiopathy, *ICH* intracerebral hemorrhage, *LAA* Large artery atherosclerosis, *MELAS* Mitochondrial encephalopathy, lactic acidosis, and stroke-like episodes, *NHLBI ESP* The National Heart, Lung, and Blood Institute, Exome Sequencing Project, *SVD* Small vessel diseases, *WES* Whole-exome sequencing

*NOTCH3* is the most prevalent disease-causing gene in our cohort of familial stroke. The existence of certain *NOTCH3* variants is also an important risk factor for sporadic stroke in the Taiwan population (Lee et al. 2019). Because *NOTCH3* p.R544C is the leading hotspot in both familial stroke (15/161, 9.3%, in this study) and sporadic stroke patients in Taiwan (Wei et al. 2021), suspicious patients with CADASIL syndrome could be screened for *NOTCH3* p.R544C by Sanger sequencing. However, if the hotspot screening turns negative, it would be tedious and time-consuming to screen the whole *NOTCH3* gene by conventional sequencing, or search for other monogenic cerebrovascular diseases*.* In this study, we adopted Taiwan Biobank (TWB) 2.0 SNP Array in the genetic diagnosis pipeline, which includes *NOTCH3* p.R544C and other pathogenic intronic and mitochondrial variants not covered by WES, like m.3243 A > G for MELAS (Wei et al. 2021). Our integrative NGS approach combining WES and TWB 2.0 Array may be an efficient alternative to conventional sequencing for non-CADASIL stroke patients. The establishment of genetic diagnosis is important for personalized antiplatelet agents (Chung et al. 2020) and early risk factor modification for the carriers of these disease-associated variants (Lee et al. 2019).

Genetic diagnosis of familial CCM is an emerging issue. Familial CCM, with the hallmark of multiple ICH of structural vasculopathy subtype, has significantly higher risks of rebleeding and epilepsy than the sporadic CCM cases. The Angioma Alliance Scientific Advisory Board Clinical Experts Panel has recommended that diagnostic genetic testing should be performed when there is a positive family history or multiple CCMs (Akers et al. 2017). The three responsible genes, *CCM1 (KRIT1), CCM2 (MGC4607)* and *CCM3 (PDCD10)*, are associated with variable risk of rebleeding or refractory epilepsy (Fischer et al. 2013; Yadla et al. 2010; Riant et al. 2010). *CCM3* variations have been reported to be associated with a higher risk of early-onset ICH and recurrent bleeding (Choquet et al. 2015; Riant et al. 2013; Shenkar et al. 2015). Therefore, the genetic etiology is essential for prediction of the risk and severity of CCM-related epilepsy or ICH and interconnected to decision of genetic testing and individualized treatment plans.

There are limitations of this study. It should be very cautious in determining the clinical significance of the identified rare variants. First, it is particularly challenging when facing the novel variants or VUS with limited clinical evidence. Accumulating genomic data in large population may help to clarify the significance of some undetermined variants (VUS in 39 out of 161, 24.2%) in future. Second, the function of the novel variant *F2* p.F382L is unknown. Further studies of the impact on thrombin-fibrin interaction and thrombin antithrombin complex (Miyawaki et al. 2012) are warranted to elucidate the pathophysiology. Third, the scope of this study primarily focused on known genes related to stroke. Given that trio study was unavailable for most of our mid-age-onset and late-onset stroke probands, there are probable novel causative genes concealed in the unidentified cases (89 out of 161, 55.3%). Fourth, severe patients could not sign the consent, e.g., unconscious or unstable vital signs, thus there was selection bias by the severity of stroke. Lastly, from our small, single center cohort, LAA and cardioembolic stroke subtypes took much smaller proportions in the patients with a definite genetic diagnosis, suggesting that these subtypes are more likely secondary to acquired and aging factors rather than monogenic causes. Further studies are warranted.

In summary, monogenic diseases cause a significant number of familial strokes, particularly in patients with the ICH or SVD subtypes. This study supported the subtype-based WES is effective in delineating rare monogenic etiologies of familial stroke. Well-characterized phenotypes, including the clinical and neuroimaging manifestations, are the cornerstone to guide the diagnostic approach and enhance the genetic yield rate. We also proposed the potential of SNP array to be an alternative to screen ethnic gene hotspots. The integrative diagnostic pipeline could target the most prevalent monogenic causes and benefit patients and their families by introducing therapeutic interventions and therefore improve the personalized stroke medicine. Accumulating knowledge of the genotype–phenotype correlations would strengthen our understanding of the genomic landscape of stroke.

## Supplementary Information

Below is the link to the electronic supplementary material.

Supplementary file1 (DOCX 960 kb)

## Data Availability

The datasets analyzed during the current study are not publicly available due to IRB restriction. The corresponding author is willing to apply for IRB permission on reasonable request.

## References

[CR1] Adams HP, Bendixen BH, Kappelle LJ, Biller J, Love BB, Gordon DL, Marsh EE (1993). Classification of subtype of acute ischemic stroke. Definitions for use in a multicenter clinical trial. TOAST Trial of Org 10172 in acute stroke treatment. Stroke.

[CR2] Akers A, Al-Shahi Salman R, Issam AA, Dahlem K, Flemming K, Hart B (2017). Synopsis of guidelines for the clinical management of cerebral cavernous malformations: consensus recommendations based on systematic literature review by the angioma alliance scientific advisory board clinical experts panel. Neurosurgery.

[CR3] Andrews S (2010) FastQC: A Quality Control Tool for High Throughput Sequence Data.

[CR4] Auer PL, Nalls M, Meschia JF, Worrall BB, Longstreth WT, Seshadri S (2015). Rare and coding region genetic variants associated with risk of ischemic stroke: the NHLBI exome sequence project. JAMA Neurol.

[CR5] Bang OY, Chung J-W, Kim DH, Won H-H, Yeon JY, Ki C-S (2020). Moyamoya disease and spectrums of RNF213 vasculopathy. Transl Stroke Res.

[CR6] Bersano A, Markus HS, Quaglini S, Arbustini E, Lanfranconi S, Micieli G (2016). Clinical pregenetic screening for stroke monogenic diseases: results from Lombardia GENS registry. Stroke.

[CR7] Carrera C, Jimenez-Conde J, Derdak S, Rabionet K, Vives-Bauza C, Soriano-Tarrega C (2016). Whole exome sequencing analysis reveals TRPV3 as a risk factor for cardioembolic stroke. Thromb Haemost.

[CR8] Choquet H, Pawlikowska L, Lawton MT, Kim H (2015). Genetics of cerebral cavernous malformations: current status and future prospects. J Neurosurg Sci.

[CR9] Chung J-W, Kim BJ, Han M-K, Kang K, Park J-M, Park S-S (2016). Family history and risk of recurrent stroke. Stroke.

[CR10] Chung CP, Chen JW, Chang FC, Li WC, Lee YC, Chen LF, Liao YC (2020). Cerebral microbleed burdens in specific brain regions are associated with disease severity of cerebral autosomal dominant arteriopathy with subcortical infarcts and leukoencephalopathy. J Am Heart Assoc.

[CR11] Dichgans M, Pulit SL, Rosand J (2019). Stroke genetics: discovery, biology, and clinical applications. Lancet Neurol.

[CR12] Fischer A, Zalvide J, Faurobert E, Albiges-Rizo C, Tournier-Lasserve E (2013). Cerebral cavernous malformations: from CCM genes to endothelial cell homeostasis. Trends Mol Med.

[CR13] Fujimura M, Tominaga T, Kuroda S, Takahashi JC, Endo H, Ogasawara K, Miyamoto S (2022). 2021 Japanese guidelines for the management of moyamoya disease: guidelines from the research committee on moyamoya disease and japan stroke society. Neurol Med Chir (tokyo).

[CR14] Gurdasani D, Barroso I, Zeggini E, Sandhu MS (2019). Genomics of disease risk in globally diverse populations. Nat Rev Genet.

[CR15] Hamosh A, Scott AF, Amberger JS, Bocchini CA, McKusick VA (2005). Online Mendelian Inheritance in Man (OMIM), a knowledgebase of human genes and genetic disorders. Nucleic Acids Res.

[CR16] Huntington JA (2005). Molecular recognition mechanisms of thrombin. J Thromb Haemost.

[CR17] Ilinca A, Kristoffersson U, Soller M, Lindgren AG (2016). Familial aggregation of stroke amongst young patients in Lund stroke register. Eur J Neurol.

[CR18] Ilinca A, Samuelsson S, Piccinelli P, Soller M, Kristoffersson U, Lindgren AG (2019). A stroke gene panel for whole-exome sequencing. Eur J Hum Genet.

[CR19] Ilinca A, Martinez-Majander N, Samuelsson S, Piccinelli P, Truvé K, Cole J (2020). Whole-exome sequencing in 22 young ischemic stroke patients with familial clustering of stroke. Stroke.

[CR20] Ioannidis NM, Rothstein JH, Pejaver V, Middha S, McDonnell SK, Baheti S (2016). REVEL: an ensemble method for predicting the pathogenicity of rare missense variants. Am J Hum Genet.

[CR21] Ishigami D, Miyawaki S, Imai H, Shimizu M, Hongo H, Dofuku S (2022). RNF213 p.Arg4810Lys heterozygosity in moyamoya disease indicates early onset and bilateral cerebrovascular events. Transl Stroke Res.

[CR23] Kalia SS, Adelman K, Bale SJ, Chung WK, Eng C, Evans JP (2017). Recommendations for reporting of secondary findings in clinical exome and genome sequencing, 2016 update (ACMG SF v2.0): a policy statement of the American College of Medical Genetics and Genomics. Genet Med.

[CR24] Kim BJ, Kim JS (2014). Ischemic stroke subtype classification: an asian viewpoint. J Stroke.

[CR25] Lee MJ, Chen YF, Fan PC, Wang KC, Wang K, Wang J, Kuo MF (2015). Mutation genotypes of RNF213 gene from moyamoya patients in Taiwan. J Neurol Sci.

[CR26] Lee YC, Chung CP, Chao NC, Fuh JL, Chang FC, Soong BW, Liao YC (2018). Characterization of heterozygous HTRA1 mutations in Taiwanese patients with cerebral small vessel disease. Stroke.

[CR27] Lee Y-C, Chung C-P, Chang M-H, Wang S-J, Liao Y-C (2019). NOTCH3 cysteine-altering variant is an important risk factor for stroke in the Taiwanese population. Neurology.

[CR28] Li H, Durbin R (2009). Fast and accurate short read alignment with Burrows-Wheeler transform. Bioinformatics.

[CR29] Li Q, Wang K (2017). InterVar: clinical interpretation of genetic variants by the 2015 ACMG-AMP guidelines. Am J Human Genet.

[CR30] Li H, Handsaker B, Wysoker A, Fennell T, Ruan J, Homer N (2009). The sequence alignment/Map format and SAMtools. Bioinformatics.

[CR31] Liao Y-C, Hsiao C-T, Fuh J-L, Chern C-M, Lee W-J, Guo Y-C (2015). Characterization of CADASIL among the Han Chinese in Taiwan: distinct genotypic and phenotypic profiles. PLoS ONE.

[CR32] McKenna A, Hanna M, Banks E, Sivachenko A, Cibulskis K, Kernytsky A (2010). The genome analysis toolkit: a MapReduce framework for analyzing next-generation DNA sequencing data. Genome Res.

[CR33] McLaren W, Gil L, Hunt SE, Riat HS, Ritchie GRS, Thormann A, Flicek P, Cunningham F (2016). The ensembl variant effect predictor. Genome Biol.

[CR34] Merello E, Pavanello M, Consales A, Mascelli S, Raso A, Accogli A, Cama A, Valeria C, De Marco P (2016). Genetic screening of pediatric cavernous malformations. J Mol Neurosci.

[CR35] Meretoja A, Strbian D, Putaala J, Curtze S, Haapaniemi E, Mustanoja S (2012). SMASH-U: a proposal for etiologic classification of intracerebral hemorrhage. Stroke.

[CR36] Miksch S, Lumsden A, Guenther UP, Foernzler D, Christen-Zäch S, Daugherty C (2005). Molecular genetics of pseudoxanthoma elasticum: type and frequency of mutations in ABCC6. Hum Mutat.

[CR37] Mishra A, Chauhan G, Violleau MH, Vojinovic D, Jian X, Bis JC (2019). Association of variants in HTRA1 and NOTCH3 with MRI-defined extremes of cerebral small vessel disease in older subjects. Brain.

[CR38] Miyawaki Y, Suzuki A, Fujita J, Maki A, Okuyama E, Murata M (2012). Thrombosis from a prothrombin mutation conveying antithrombin resistance. N Engl J Med.

[CR39] Mukawa M, Nariai T, Onda H, Yoneyama T, Aihara Y, Hirota K (2017). Exome sequencing identified CCER2 as a novel candidate gene for moyamoya disease. J Stroke Cerebrovasc Dis.

[CR40] Plomp AS, Hu X, de Jong PTVM, Bergen AAB (2004). Does autosomal dominant pseudoxanthoma elasticum exist?. Am J Med Genet Part A.

[CR41] Rabbani B, Tekin M, Mahdieh N (2014). The promise of whole-exome sequencing in medical genetics. J Hum Genet.

[CR42] Riant F, Bergametti F, Ayrignac X, Boulday G, Tournier-Lasserve E (2010). Recent insights into cerebral cavernous malformations: the molecular genetics of CCM. FEBS J.

[CR43] Riant F, Bergametti F, Fournier HD, Chapon F, Michalak-Provost S, Cecillon M (2013). CCM3 mutations are associated with early-onset cerebral hemorrhage and multiple meningiomas. Mol Syndromol.

[CR44] Santoro C, Giugliano T, Kraemer M, Torella A, Schwitalla JC, Cirillo M (2018). Whole exome sequencing identifies MRVI1 as a susceptibility gene for moyamoya syndrome in neurofibromatosis type 1. PLoS ONE.

[CR45] Sauvigny T, Alawi M, Krause L, Renner S, Spohn M, Busch A (2020). Exome sequencing in 38 patients with intracranial aneurysms and subarachnoid hemorrhage. J Neurol.

[CR46] Shenkar R, Shi C, Rebeiz T, Stockton RA, McDonald DA, Mikati AG (2015). Exceptional aggressiveness of cerebral cavernous malformation disease associated with PDCD10 mutations. Genet Med.

[CR47] Starby H, Delavaran H, Andsberg G, Lovkvist H, Norrving B, Lindgren A (2014). Multiplicity of risk factors in ischemic stroke patients: relations to age, sex, and subtype–a study of 2,505 patients from the lund stroke register. Neuroepidemiology.

[CR48] Takagi Y, Murata M, Kozuka T, Nakata Y, Hasebe R, Tamura S (2016). Missense mutations in the gene encoding prothrombin corresponding to Arg596 cause antithrombin resistance and thrombomodulin resistance. Thromb Haemost.

[CR49] Tan RYY, Traylor M, Megy K, Duarte D, Deevi SVV, Shamardina O (2019). How common are single gene mutations as a cause for lacunar stroke?. Neurology.

[CR50] Toyoda K, Koga M, Hayakawa M, Yamagami H (2015). Acute reperfusion therapy and stroke care in Asia after successful endovascular trials. Stroke.

[CR51] Van der Auwera GA, Carneiro MO, Hartl C, Poplin R, Del Angel G, Levy-Moonshine A (2013). From FastQ data to high confidence variant calls: the genome analysis toolkit best practices pipeline. Curr Protoc Bioinformatics.

[CR52] Wei C-Y, Yang J-H, Yeh E-C, Tsai M-F, Kao H-J, Lo C-Z (2021). Genetic profiles of 103,106 individuals in the Taiwan Biobank provide insights into the health and history of Han Chinese. Npj Genomic Med.

[CR53] Yadla S, Jabbour PM, Shenkar R, Shi C, Campbell PG, Awad IA (2010). Cerebral cavernous malformations as a disease of vascular permeability: from bench to bedside with caution. Neurosurg Focus.

[CR54] Yaghi S, Shu L, Bakradze E, Omran SS, Giles JA, Amar JY (2022). Direct oral anticoagulants versus warfarin in the treatment of cerebral venous thrombosis (ACTION-CVT): a multicenter international study. Stroke.

