# Supplementary material for: Monogenic Causes in Familial Stroke Across Intracerebral Hemorrhage and Ischemic Stroke Subtypes Identified by Whole-Exome Sequencing
Source: Cell Mol Neurobiol. 2022 Dec 29;43(6):2769–83. doi: 10.1007/s10571-022-01315-3 (PMC10333419; doi:10.1007/s10571-022-01315-3)
Supplement: Supplementary file 1 — Supplementary file1 (DOCX 960 kb) [file 10571_2022_1315_MOESM1_ESM.docx]

**SUPPLEMENTAL MATERIAL**

**Monogenic causes in familial stroke across intracerebral hemorrhage and ischemic stroke subtypes identified by whole exome sequencing**

Li-Hsin Chang^1^, BS; Nai-Fang Chi^2,3^, MD, PhD; Chun-Yu Chen^2^, MD, PhD; Yung-Shuan Lin^2^, MD; Shao-Lun Hsu^2^, MD; Jui-Yao Tsai, MS^2^; Hui-Chi Huang, MS^2^; Chun-Jen Lin^2,3^, MD, PhD; Chih-Ping Chung^2,3^, MD, PhD; Chien-Yi Tung, PhD^5^; Chung-Jiuan Jeng, PhD^4,6^; Yi-Chung Lee, MD, PhD^2,3,4^; Yo-Tsen Liu, MD, PhD^1,2,3,4 *^; I-Hui Lee^2,3,4 *^, MD, PhD

^a^Institute of Brain Science, National Yang Ming Chiao Tung University, Taipei, Taiwan

^b^Department of Neurology, Neurological Institute, Taipei Veterans General Hospital, Taipei, Taiwan

^c^School of Medicine, National Yang Ming Chiao Tung University, Taipei, Taiwan

^d^Brain Research Center, National Yang Ming Chiao Tung University, Taipei, Taiwan

^e^Cancer Progression Research Center, National Yang Ming Chiao Tung University, Taipei, Taiwan.

^f^Institute of Anatomy and Cell Biology, School of Medicine, National Yang Ming Chiao Tung University, Taipei, Taiwan.

**Corresponding authors**:

Yo-Tsen Liu, MD, PhD

Address: No.201, Sec. 2, Shipai Rd., Beitou District, Taipei City, Taiwan 11217

Phone: +886-2-28712121

Fax: +886-2-28757579

E-mail address: [ytliu2@vghtpe.gov.tw](mailto:ytliu2@vghtpe.gov.tw)

ORCID: 0000-0001-5902-3870

I-Hui Lee, MD, PhD

Address: No.201, Sec. 2, Shipai Rd., Beitou District, Taipei City, Taiwan 11217

Phone: +886-2-28712121 ext.8109

Fax: +886-2-28757579

E-mail address: [ihlee@vghtpe.gov.tw](mailto:ihlee@vghtpe.gov.tw)

ORCID: 0000-0002-5344-6685

**Table S1: Candidate stroke gene list**

| *ABCA1* | *ABCC6* | *ABCG5* | *ABCG8* | *ACAD9* | *ACP5* | *ACTA2* | *ACTB* |
| --- | --- | --- | --- | --- | --- | --- | --- |
| *ACTC1* | *ACTG1* | *ACVRL1* | *ADA2* | *ADAMTS13* | *ADIPOQ* | *AGXT* | *AKT1* |
| *ALOX5AP* | *ANGPTL6* | *APC* | *APOA1* | *APOB* | *APP* | *ASS1* | *ATP7A* |
| *ATP7B* | *B3GALT6* | *B4GALT1* | *BAP1* | *BAZ1B* | *BMPR1A* | *BRCA1* | *BRCA2* |
| *BRCC3* | *C1R* | *CACNA1A* | *CACNA1S* | *CALR* | *CBS* | *CCM2* | *CD46* |
| *CD59* | *CDK18* | *CFH* | *CFI* | *CITED2* | *CLIP2* | *CLO4A1* | *COG6* |
| *COL1A1* | *COL3A1* | *COL4A2* | *COL5A1* | *COL5A2* | *COLGALT1* | *COQ8A* | *COX1* |
| *COX2* | *CPS1* | *CPT2* | *CRELD1* | *CST3* | *CTSA* | *CUL3* | *CYP11B1* |
| *CYTB* | *DIAPH1* | *DLD* | *DLST* | *DNMT3A* | *DPM1* | *DPM3* | *DSC2* |
| *DSG2* | *DSP* | *DYRK1B* | *EFEMP2* | *ELN* | *ENPP1* | *EPAS1* | *EPOR* |
| *ERCC6* | *ERCC8* | *ESCO2* | *F10* | *F13A1* | *F13B* | *F2* | *F5* |
| *F7* | *F8A1* | *F9* | *FBLN5* | *FBN1* | *FCGR2C* | *FERMT3* | *FGA* |
| *FGB* | *FGG* | *FH* | *FLNA* | *FLNC* | *FN1* | *FOXC1* | *FOXE3* |
| *GAA* | *GATA3* | *GATA4* | *GATA6* | *GCDH* | *GDF2* | *GFI1B* | *GFND1* |
| *GGCX* | *GLA* | *GNAQ* | *GNDF* | *GP1BA* | *GPR143* | *GTF2I* | *GTF2IRD1* |
| *GUCY1A1* | *GYS1* | *HBB* | *HCFC2* | *HELLPAR* | *HHT4* | *HSD11B2* | *HTRA1* |
| *IL1RN* | *ISCU* | *ITM2B* | *IVD* | *JAG1* | *JAK2* | *JAM3* | *KCNA5* |
| *KCNE2* | *KCNH2* | *KCNJ2* | *KCNQ1* | *KIF1B* | *KIF20A* | *KLHL3* | *KNG1* |
| *KRAS* | *KRIT1* | *LDLR* | *LIMK1* | *LMNA* | *LOX* | *LYL1* | *MAT2A* |
| *MAX* | *MBTPS2* | *MDH2* | *MECP2* | *MEN1* | *MFAP5* | *MFN2* | *MGAT2* |
| *MLH1* | *MLXIPL* | *MMACHC* | *MMUT* | *MPI* | *MPL* | *MSH2* | *MSH6* |
| *MTHFR* | *MUTYH* | *MYBPC3* | *MYD88* | *MYH11* | *MYH6* | *MYH7* | *MYH9* |
| *MYL2* | *MYL3* | *MYLK* | *MYMY4* | *MYPN* | *NAGS* | *NBEAL2* | *NCF1* |
| *ND1* | *ND4* | *ND5* | *ND6* | *NDE1* | *NDUFA8* | *NF1* | *NF2* |
| *NKX2-5* | *NOS3* | *NOTCH1* | *NOTCH3* | *NPPA* | *NR2D2* | *NR3C1* | *NR3C2* |
| *NUP155* | *OTC* | *PCCA* | *PCCB* | *PCNT* | *PCSK9* | *PDCD10* | *PDE3A* |
| *PDE4D* | *PDGFB* | *PGM1* | *PHACTR1* | *PIGA* | *PIK3C2A* | *PIK3CA* | *PKD1* |
| *PKP2* | *PLG* | *PLOD1* | *PLOD3* | *PMM2* | *PMS2* | *PNP* | *PRKAG2* |
| *PRKCH* | *PRKG1* | *PRNP* | *PROC* | *PROS1* | *PTEN* | *PTPN11* | *RASA1* |
| *RASGRP2* | *RB1* | *RET* | *RFC2* | *RFT1* | *RNF213* | *SAG* | *SAMHD1* |
| *SCN1B* | *SCN2B* | *SCN3B* | *SCN4B* | *SCN5A* | *SCNN1B* | *SCNN1G* | *SDHA* |
| *SDHAF2* | *SDHB* | *SDHC* | *SDHD* | *SERPINC1* | *SERPINE1* | *SH2B3* | *SLC19A2* |
| *SLC25A11* | *SLC2A10* | *SMAD3* | *SMAD4* | *SMAD9* | *SMARCAL1* | *SMARCB1* | *SMARCE1* |
| *SMO* | *SNAP29* | *SON* | *SPARC* | *STAT1* | *STAT2* | *STIM1* | *STK11* |
| *STX1A* | *SUFU* | *TANGO2* | *TBL2* | *TBX20* | *TENT5A* | *TERT* | *TET2* |
| *TGFB2* | *TGFB3* | *TGFBR1* | *TGFBR2* | *TGFBR3* | *THBD* | *THPO* | *THSD1* |
| *TLL1* | *TMEM127* | *TMEM43* | *TNNI3* | *TNNI3TNNT2* | *TNNT2* | *TNXB* | *TP53* |
| *TPM1* | *TPP2* | *TRAF7* | *TREM2* | *TREX1* | *TRNC* | *TRNF* | *TRNH* |
| *TRNK* | *TRNL1* | *TRNQ* | *TRNS1* | *TRNS2* | *TRNV* | *TRNW* | *TSC1* |
| *TSC2* | *TTR* | *USP18* | *VHL* | *VPS52* | *VWF* | *WFS1* | *WT1* |
| *XYLT1* | *XYLT2* | *YY1AP1* | *ZAP70* | *ZMPSTE24* |  |  |  |

Totally 325 stroke-related genes selected according to the Online Mendelian Inheritance in Man database, literature review, and 7 Human Phenotype Ontology terms including stroke, ischemic stroke, hemorrhage stroke, stroke-like episode, cerebral hemorrhage, lacunar stroke, and thromboembolic stroke.

**Figure S1: Fourteen pedigrees of *NOTCH3:* p.R544C pathogenic variants**

**
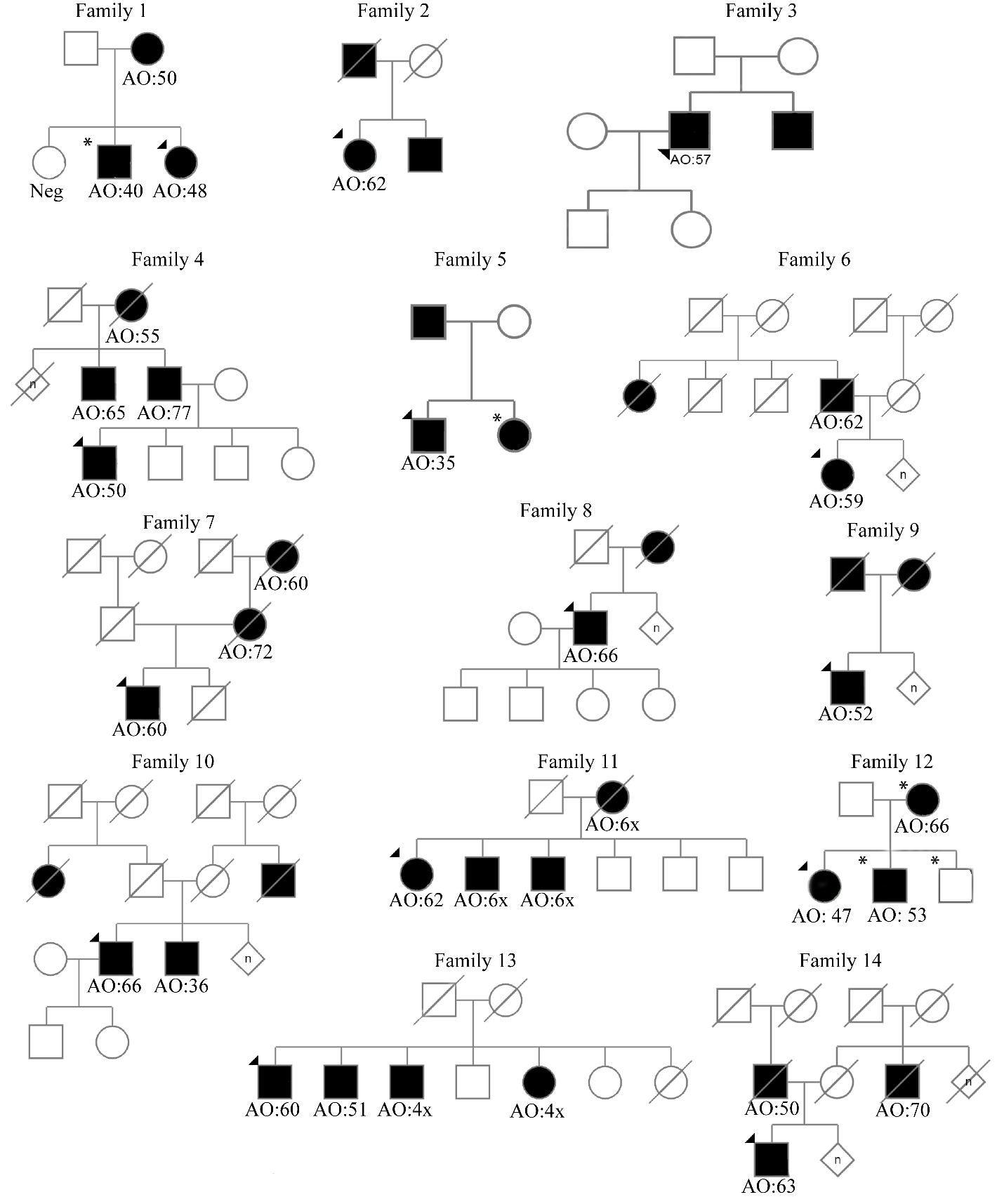
**

We identified 33 families carried pathogenic or likely pathogenic variants. Among them, 15 families identified carried *NOTCH3*: p.R544C (Another 1 pedigree shown in figure 4c) and 6 of their family members also confirmed the same genotype. The average age of onset of stroke was 55.9 ± 10.4. * Phenotype and genotype segregated family members.

**Figure S2: Pedigrees of other families carrying pathogenic or likely pathogenic variants**


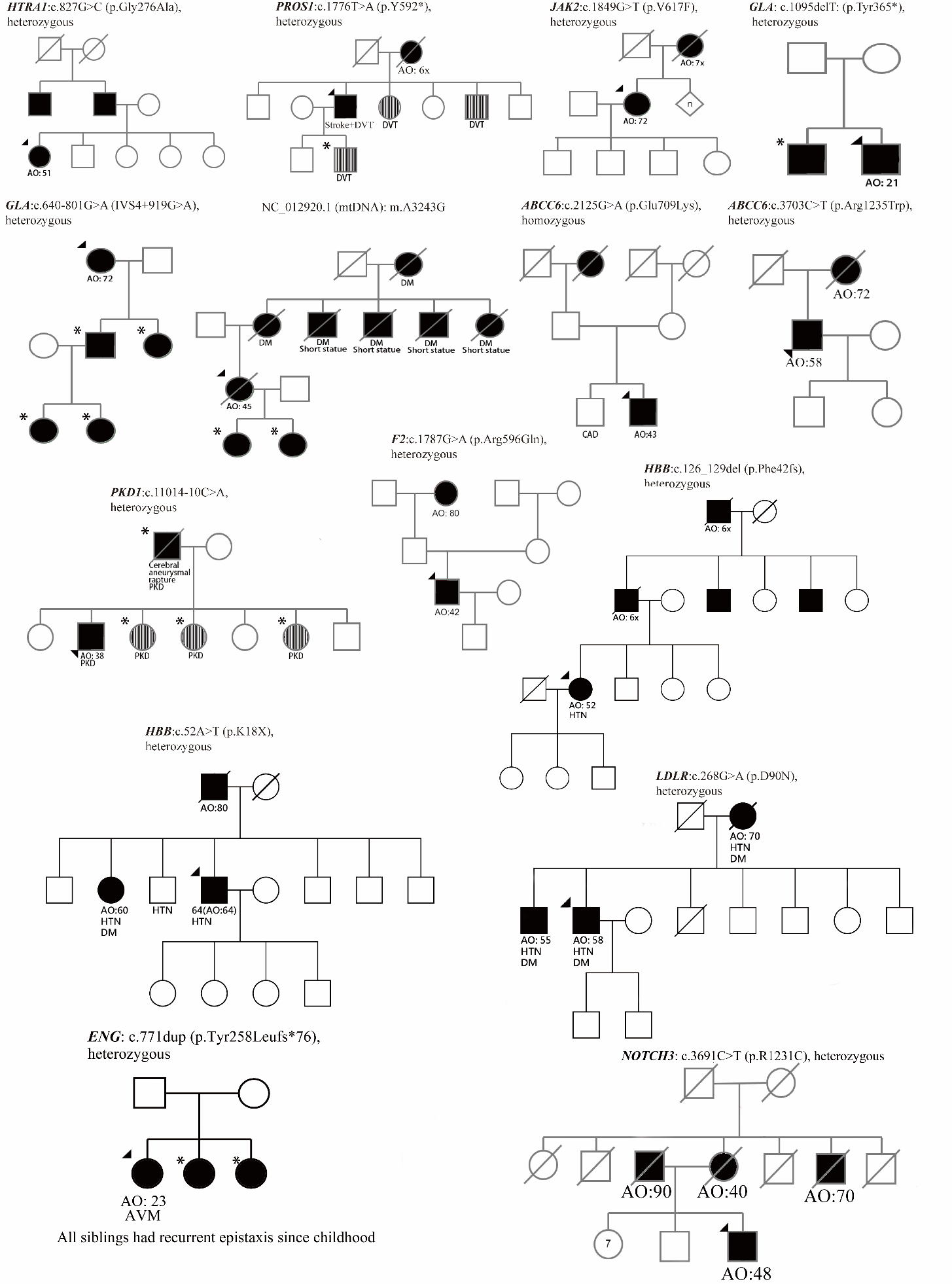


Another 18 families identified with monogenic stroke (3 pedigrees shown in figure 4a, 4b and 4d), including *HBB* (n=2)*, F2* (n=2), *ABCC6* (n=2)*, GLA* (n=2), ENG, *HTRA1, PROS1, JAK2,* m.3243A>G*, RNF213, LDLR, PKD1, NOTCH3, and KRIT1*. Seventeen family members, including *GLA* (n=5), *PKD1* (n=4), m.3243A>G (n=2), *ENG* (n=2), *F2* (n=2), *PROS1* (n=1), and *KRIT1* (n=1), segregated with the probands’ variants. * Phenotype and genotype segregated family members.
